# Cardiovascular profiling in the diabetic continuum: results from the population-based Gutenberg Health Study

**DOI:** 10.1007/s00392-021-01879-y

**Published:** 2021-06-24

**Authors:** Volker H. Schmitt, Anja Leuschner, Claus Jünger, Antonio Pinto, Omar Hahad, Andreas Schulz, Natalie Arnold, Sven-Oliver Tröbs, Marina Panova-Noeva, Karsten Keller, Tanja Zeller, Manfred Beutel, Norbert Pfeiffer, Konstantin Strauch, Stefan Blankenberg, Karl J. Lackner, Jürgen H. Prochaska, Philipp S. Wild, Thomas Münzel

**Affiliations:** 1 Department of Cardiology – Cardiology I, University Medical Center, Johannes Gutenberg University Mainz, Langenbeckstr. 1, 55131 Mainz, Germany; 2 German Center for Cardiovascular Research (DZHK), Partner Site Rhine-Main, Mainz, Germany; 3 Preventive Cardiology and Preventive Medicine – Department of Cardiology, University Medical Center, Johannes Gutenberg University Mainz, Langenbeckstr. 1, 55131 Mainz, Germany; 4 Center for Thrombosis and Hemostasis (CTH), University Medical Center, Johannes Gutenberg University Mainz, Langenbeckstr. 1, 55131 Mainz, Germany; 5 Department of General and Interventional Cardiology, University Heart Center Hamburg, Martinistr 52, 20246 Hamburg, Germany; 6 German Center for Cardiovascular Research (DZHK), Partner Site Hamburg/Kiel/Lübeck, Hamburg, Germany; 7 Department of Psychosomatic Medicine and Psychotherapy, University Medical Center, Johannes Gutenberg University Mainz, Langenbeckstr. 1, 55131 Mainz, Germany; 8 Department of Ophthalmology, University Medical Center, Johannes Gutenberg University Mainz, Langenbeckstr. 1, 55131 Mainz, Germany; 9 Institute for Medical Biometrics, Epidemiology and Informatics (IMBEI), University Medical Center, Johannes Gutenberg University Mainz, Obere Zahlbacher Str. 69, 55131 Mainz, Germany; 10 Institute of Clinical Chemistry and Laboratory Medicine, University Medical Center, Johannes Gutenberg University Mainz, Langenbeckstr. 1, 55131 Mainz, Germany; 11 Medical Clinic VII, Department of Sports Medicine, University Hospital Heidelberg, Heidelberg, Germany

**Keywords:** Prediabetes, Type 2 diabetes mellitus, Cardiovascular disease, Asymptomatic organ damage, All-cause mortality, Disease prevention

## Abstract

**Aims:**

To assess the prevalence of type 2 diabetes mellitus (T2DM) and prediabetes in the general population and to investigate the associated cardiovascular burden and clinical outcome.

**Methods and Results:**

The study sample comprised 15,010 individuals aged 35–74 years of the population-based Gutenberg Health Study. Subjects were classified into euglycaemia, prediabetes and T2DM according to clinical and metabolic (HbA1c) information. The prevalence of prediabetes was 9.5% (*n* = 1415) and of T2DM 8.9% (*n* = 1316). Prediabetes and T2DM showed a significantly increased prevalence ratio (PR) for age, obesity, active smoking, dyslipidemia, and arterial hypertension compared to euglycaemia (for all, *P* < 0.0001). In a robust Poisson regression analysis, prediabetes was established as an independent predictor of clinically-prevalent cardiovascular disease (PR_prediabetes_ 1.20, 95% CI 1.07–1.35, *P* = 0.002) and represented as a risk factor for asymptomatic cardiovascular organ damage independent of traditional risk factors (PR 1.04, 95% CI 1.01–1.08, *P* = 0.025). Prediabetes was associated with a 1.5-fold increased 10-year risk for cardiovascular disease compared to euglycaemia. In Cox regression analysis, prediabetes (HR 2.10, 95% CI 1.76–2.51, *P* < 0.0001) and T2DM (HR 4.28, 95% CI 3.73–4.92, *P* < 0.0001) indicated for an increased risk of death. After adjustment for age, sex and traditional cardiovascular risk factors, only T2DM (HR 1.89, 95% CI 1.63–2.20, *P* < 0.0001) remained independently associated with increased all-cause mortality.

**Conclusion:**

Besides T2DM, also prediabetes inherits a significant cardiovascular burden, which translates into poor clinical outcome and indicates the need for new concepts regarding the prevention of cardiometabolic disorders.

**Supplementary Information:**

The online version contains supplementary material available at 10.1007/s00392-021-01879-y.

## Introduction

Type 2 diabetes mellitus (T2DM) is the leading metabolic disease worldwide affecting approximately 422 million adults according to the World Health Organisation [1]. T2DM represents a well-established risk factor for cardiovascular disease (CVD) and inherits a major public health burden [2]. Besides the epidemic of T2DM, a high prevalence of prediabetes, which confers a state of dysregulated glucose homeostasis predisposing to the development of T2DM, was reported. Importantly, it is estimated that 470 million people worldwide will suffer from prediabetes by 2030 [3]. In addition, prediabetes seems already to be accompanied by an elevated risk for the development of (sub)clinical CVD [4].

In the literature, evidence is available that already early phases of dysregulated glucose metabolism might be associated with an elevated risk for the development of CVD [5] by promoting atherosclerosis via both a direct effect on the arterial wall and indirectly via effects on lipids and blood pressure [6]. In a recent study, we demonstrated that cardiovascular damages may precede diabetes mellitus development by showing that microvascular endothelial dysfunction is a strong independent predictor of incident prediabetes and T2DM [7]. The aim of the present study was therefore to determine the prevalence of T2DM in comparison to prediabetes in the general population using a large population-based study cohort, including more than 15,000 people, to evaluate their relation with cardiovascular risk factors (CVRF), as well as with clinical and subclinical CVD.

## Methods

### Study design

The Gutenberg Health Study is a large population-based, prospective, observational cohort study in Mid-Western Germany. The rationale and design of the study has been published recently [8]. Between 2007 and 2011, a total of 15,010 individuals underwent a highly standardised investigational plan at the study platform. The study was approved by the local ethics committee (reference no. 837.020.07[5555]) and the data protection officer before study initiation. Study participants provided written informed consent before study enrollment. All study procedures have been performed in line with the principles outlined in the Declaration of Helsinki and the recommendations for Good Clinical and Epidemiological Practice.

### Data assessment

During the 5-h investigation at the study platform, all participants underwent a highly standardised assessment, including inter alia anthropometrics, measurements of traditional CVRF, clinical comorbidities, medication, and venous blood sampling. Traditional CVRF included age, sex, arterial hypertension, diabetes mellitus, dyslipidaemia, obesity, smoking, and positive family history of myocardial infarction or stroke (see Supplemental Table S1 for further information). CVD was assessed in a standardised computer-assisted personal interview as composite of the following diseases being diagnosed by a physician: coronary artery disease, myocardial infarction, stroke or transient ischemic attack, heart failure, atrial fibrillation, and peripheral artery disease. In individuals without prevalent CVD, asymptomatic cardiovascular organ damage (AOD) as indicator of early, subclinical CVD was assessed according to current guideline recommendations [9]. In brief, the following variables were applied as indicators for presence of asymptomatic organ damage in the study population: elevated left ventricular mass index (men > 115 g/m^2^, women > 95 g/m^2^), carotid intima-media-thickness > 0.9 mm, presence of at least one atherosclerotic plaque in the carotid artery, ankle–brachial-index < 0.9, moderate renal insufficiency (i.e. estimated glomerular filtration rate 30–60 ml/min/1.73 m^2^ according to the Chronic Kidney Disease Epidemiology Collaboration formula, subjects with severe renal insufficiency defined by an estimated glomerular filtration rate < 30 ml/min/1.73 m^2^ were not included), or microalbuminuria (albumin–creatinine-ratio 30–300 mg/g) (see Supplemental Table S2 for further information).

### Definitions for phenotypes of the glucose metabolism

Euglycaemia, prediabetes and T2DM were defined in line with current recommendations of the National Institute for Health and Care Excellence and the International Expert Committee [10, 11]. Concentrations of HbA1c were determined under highly standardised conditions within the daily clinical routine diagnostics at the Institute of Clinical Chemistry and Laboratory Medicine of the University Medical Centre Mainz using a high-performance liquid chromatography assay (Bio-Rad Laboratories, Hercules, California, USA).

According to a predefined algorithm incorporating clinical and molecular information, the diabetic phenotypes were defined as follows: Individuals with HbA1c ≥ 6.5% (≥ 48 mmol/mol), T2DM diagnosed by a physician or intake of antidiabetic drugs were categorised as T2DM. Prediabetes was recorded for individuals with HbA1c between 6.0% and 6.4% (42–47 mmol/mol). Individuals with HbA1c levels < 6.0% were categorised as euglycaemic. Subjects with diabetes mellitus other than T2DM (e.g. type 1 diabetes mellitus, gestational diabetes mellitus, diabetes mellitus following pancreatitis) as well as people with euglycaemic HbA1c levels and fasting glucose > 125 mg/dl were excluded from the analysis.

Currently, two different HbA1c windows exist to define prediabetes, the one before mentioned with a range of HbA1c between 6.0 and 6.4%, and the definition given by the American Diabetes Association (ADA) defining prediabetes with HbA1c values between 5.7 and 6.4% (39–47 mmol/mol) [12]. To facilitate the comparisons between the results of the present investigation with studies using the definition of the ADA, data were additionally calculated with the HbA1c window of 5.7–6.4% for definition of prediabetes. Tables and figures providing these results are presented in the Supplemental Material.

### Statistical analyses

Continuous variables were described by mean and standard deviation (SD) or for skewed distribution (defined as |skewness| > 1) by median and interquartile range. Categorical variables were described by relative and absolute frequencies. Fisher’s exact or Chi-squared tests were used for statistical comparison of categorical and Mann–Whitney *U* test or Student’s t test for continuous variables, as appropriate. The prevalence for phenotypes of glucose metabolism were weighted for sex and age using 5-year groups for (i) the regional population of Mainz and Mainz-Bingen, (ii) the population of Germany and (iii) the European standard population of 1976.

To investigate the relation of diabetic phenotype and CVRF, AOD and CVD, Poisson regression models with variance estimates for prevalence ratios (PR) were calculated. All models were adjusted for sex, age, and traditional CVRF (except diabetes mellitus) as potential confounders for the relation of diabetic phenotype and CVD. Since established diabetes mellitus itself is rated as equivalent of CVD, the analysis for AOD was restricted to individuals with prediabetes. To assess the cardiovascular risk of prediabetes and T2DM, the 10-year risk for incident coronary heart disease according to Framingham risk score was calculated [13]. Survival analyses were conducted by means of Kaplan–Meier curves and Cox regression models. In addition, competing risk analyses were performed for prediabetes and T2DM with all-cause death as competing risk investigating cardiac death, cardiovascular disease, myocardial infarction, stroke and atrial fibrillation as well as for the combination of cardiac death and myocardial infarction, cardiac death and stroke, cardiac death and atrial fibrillation as well as cardiac death and venous thromboembolism. *P* values < 0.05 were defined as relevant associations. All statistical analyses were performed using the statistical software package R, version 3.6.0 (R Foundation for Statistical Computing, Vienna, Austria).

## Results

### Prevalence of diabetic phenotypes in the general population

**Fig. 1:**
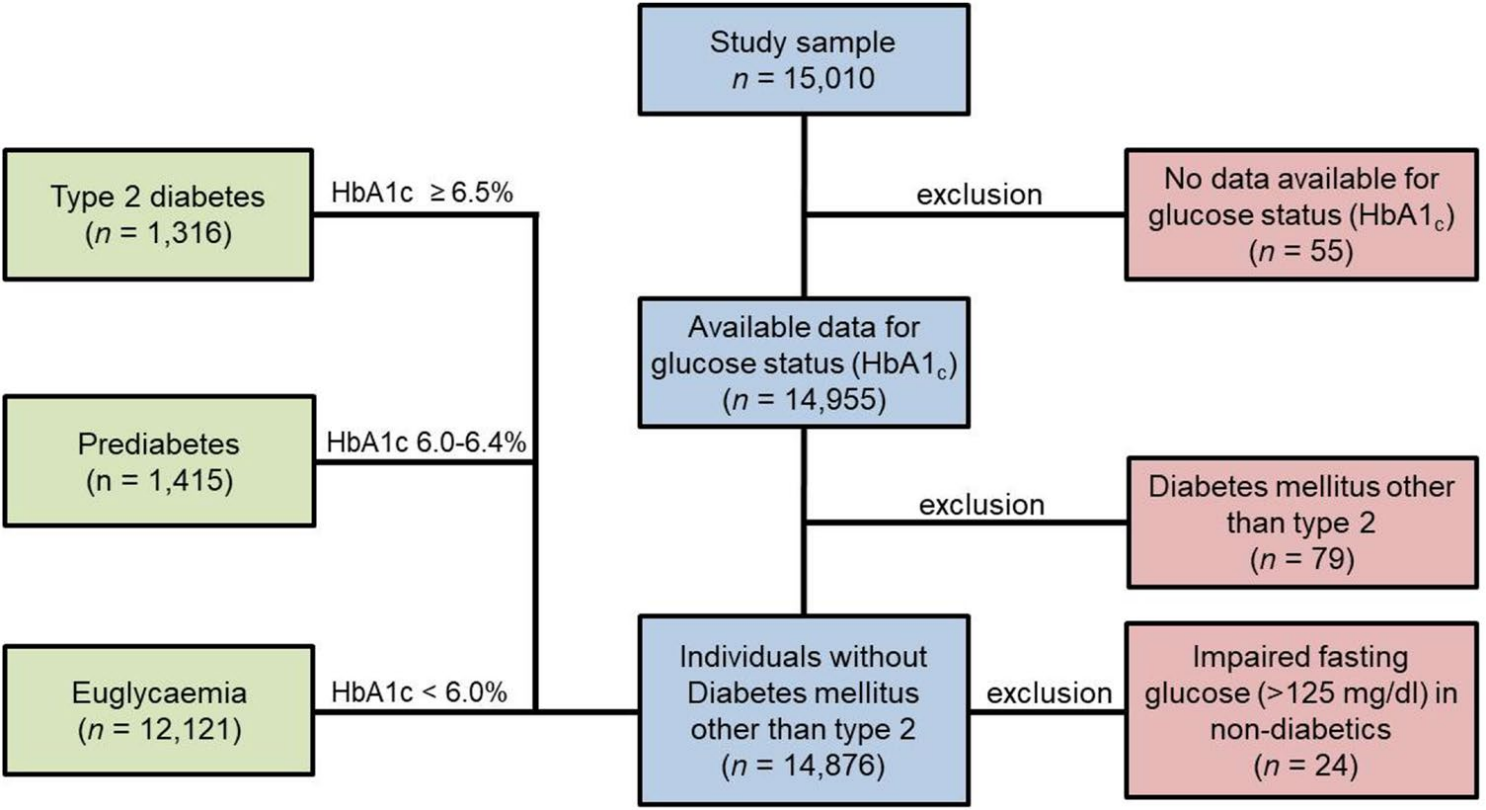
Study flow chart. T2DM was defined as HbA1c ≥ 6.5% or diagnosed by a physician or diabetic medication intake. Prediabetes was present if HbA1c 6.0–6.4% and no T2DM diagnosed by a physician and no diabetic medication intake. Euglycaemia was present if HbA1c < 6.0% and no T2DM diagnosed by a physician and no diabetic medication intake. Individuals without available data for glucose state and all diabetes types other than T2DM were excluded. Also, individuals of non-fasting state presenting fasting glucose > 125 mg/dl were excluded. Exclusions from the GHS cohort are given in red boxes. In total, 14,852 individuals were included. *GHS* Gutenberg Health Study, *T2DM* Type 2 diabetes mellitus

From the total sample of 15,010 study participants, 158 subjects were excluded due to missing information on glucose status (HbA1c), diabetes mellitus other than type 2 or impaired fasting glucose levels in no diabetic individuals. Thus, 14,852 individuals remained and were included in the present assessment. Euglycaemia was observed in 12,121 individuals, whereas prediabetes was detected in 1415 subjects and T2DM was present in 1316 individuals (Fig. 1).

The prevalence of prediabetes and T2DM increased with age within the Gutenberg Health Study (GHS) cohort. Whereas in the age group of 35 to 44 years the prevalence of prediabetes was nearly double as high as of T2DM, the gap waned and in the age decade of 55 to 64 years the prevalence of both disorders was about even. In the oldest age decade (65–74 years) more people suffered from T2DM than from prediabetes (Table 1A). The prevalence of prediabetes and T2DM in the general population of Mainz and Mainz-Bingen in mid-western Germany was ranging from 8.4% for prediabetes and 7.4% for T2DM, which was lower compared to the population of Germany (prediabetes 8.7%, T2DM 7.8%) and higher than the European standard cohort (prediabetes 8.1%, T2DM 7.0%) (Table 1B).

**Table 1:** Prevalence of euglycaemia, prediabetes and T2DM according to age within the GHS and region of residence

	35–44 years	45–54 years	55–64 years	65–74 years
A. Prevalence of prediabetes and T2DM according to age decades in the GHS study cohort				
Euglycaemia	96.4% (*n* = 3142)	88.6% (*n* = 3497)	76.9% (*n* = 3031)	66.2% (*n* = 2451)
Prediabetes	2.3% (*n* = 75)	7.0% (*n* = 277)	11.7% (*n* = 463)	16.2% (*n* = 600)
Diabetes	1.3% (*n* = 44)	4.3% (*n* = 171)	11.3% (*n* = 447)	17.7% (*n* = 654)

	GHS Study sample	Population of Mainz and Mainz-Bingen, Germany	Population of Germany	European standard population
B. Prevalence of prediabetes and T2DM in the GHS study sample and weighted for the population of Mainz/Mainz-Bingen, the population of Germany and the European standard population of 1976				
Euglycaemia	81.6% (*n* = 12,121)	84.2% (*n* = 12,505)	83.5% (*n* = 12,404)	85.0% (*n* = 12,625)
Prediabetes	9.5% (*n* = 1415)	8.4% (*n* = 1250)	8.7% (*n* = 1299)	8.1% (*n* = 1197)
Diabetes	8.9% (*n* = 1316)	7.4% (*n* = 1102)	7.8% (*n* = 1154)	7.0% (*n* = 1037)

A. Presentation of the prevalence estimates of euglycaemia, prediabetes and T2DM according to age decades in the GHS study sample. B. Table showing prevalences of euglycaemia, prediabetes and T2DM as unweighted data for the GHS study sample and weighted data (for age- and sex-distribution) for the population of Mainz/Mainz-Bingen, Germany and the European standard population of 1976. Prevalence estimates are provided as relative (%) and absolute (*n*) frequency

*GHS* Gutenberg Health Study, *T2DM* type 2 diabetes mellitus

Prevalences of prediabetes by ADA definition and T2DM in the GHS cohort as well as weighted for the general population of Mainz and Mainz-Bingen, Germany, the population of Germany and the European standard cohort are given in Supplemental Table S3.

### Diabetic phenotype and cardiovascular risk factors

Individuals with prediabetes were younger than people with T2DM, whereas the largest difference was present between euglycaemic subjects and the other two entities. Traditional CVRF were distinctly more prevalent in prediabetics compared to euglycaemics, while in people with T2DM a further marked increase was present. By this, prevalence of dyslipidaemia, hypertension and obesity were about 1.5-fold increased in prediabetes and approximately doubled in T2DM compared to euglycaemic individuals. Only smoking was highest in prediabetes and least in T2DM. Moreover, T2DM and also prediabetes were associated with considerably increased prevalence of comorbidities like congestive heart failure, coronary artery disease, myocardial infarction, peripheral artery disease and stroke (Table 2).

**Table 2:** **C**haracteristics of study participants according to diabetic status

	Euglycaemia (*n* = 12,121)	Prediabetes (*n* = 1415)	Diabetes (*n* = 1316)	*P *value
Age, mean (SD), years	53.4 ± 11.0	61.1 ± 9.0	63.0 ± 8.3	< 0.0001
Female sex	50.4% (6,110)	52.0% (736)	38.2% (503)	< 0.0001
BMI, median (IQR), kg/m^2^	26.1 (23.5/29.2)	28.4 (25.4/32.0)	30.7 (27.3/34.6)	< 0.0001
Traditional cardiovascular risk factors				
Current smoking	19.3% (2336)	23.8% (336)	16.1% (210)	0.36
Dyslipidaemia	29.9% (3616)	49.2% (695)	61.8% (810)	< 0.0001
Family history of MI and/or stroke	21.0% (2546)	26.5% (375)	27.5% (362)	< 0.0001
Hypertension	44.5% (5386)	65.9% (932)	80.2% (1,056)	< 0.0001
Obesity	20.4% (2477)	37.5% (530)	55.3% (727)	< 0.0001
Cardiovascular comorbidities				
Atrial fibrillation	2.3% (273)	3.9% (55)	5.8% (76)	< 0.0001
Congestive heart failure	1.0% (121)	2.2% (31)	3.4% (44)	< 0.0001
Coronary artery disease	2.9% (354)	7.2% (102)	13.5% (177)	< 0.0001
Myocardial infarction	1.9% (236)	5.3% (75)	9.5% (125)	< 0.0001
Peripheral artery disease	2.6% (313)	5.1% (72)	8.1% (107)	< 0.0001
Stroke	1.4% (166)	2.9% (41)	4.9% (64)	< 0.0001
Laboratory parameters of glucose metabolism				
Glucose, median (IQR), mg/dl	90.0 (85.0/95.0)	97.0 (91.0/104.0)	112.2 (99.0/131.2)	< 0.0001
Glucose (fasting), median (IQR), mg/dl	91.0 (85.0/97.0)	98.0 (92.0/105.0)	117.2 (103.0/139.0)	< 0.0001
Glucose (non-fasting), median (IQR), mg/dl	89.0 (84.0/94.0)	94.0 (89.2/100.0)	102.0 (93.9/117.0)	< 0.0001
Fasting period ≥ 8 h	70.7% (8567)	72.6% (1027)	68.5% (901)	0.37
HbA1c, median (IQR), %	5.40 (5.10/5.60)	6.10 (6.00/6.20)	6.70 (6.30/7.20)	< 0.0001

Discrete variables are expressed as relative and absolute frequencies; continuous variables are provided according to distribution as mean with standard deviation or median with interquartile range

*BMI* body mass index, *HbA1c* glycated haemoglobin, *IQR* interquartile range, *MI* myocardial infarction, *SD* standard deviation

To further investigate the link between CVRF and diabetic phenotype, robust Poisson regression analyses were conducted. Compared to T2DM, individuals with prediabetes more often were smoker (prevalence ratio (PR) 1.83, 95% confidence interval (CI) 1.63–2.06, *P* < 0.0001) as well as female (PR 1.14, 95% CI 1.04–1.26, *P* < 0.0001). In contrast, T2DM was associated with male sex (PR_female sex_ 0.76, 95% CI 0.68–0.84, *P* < 0.0001), older age (PR 1.85, 95% CI 1.75–1.96, *P* < 0.0001) and higher presence of dyslipidaemia (PR 1.88, 95% CI 1.69–2.09, *P* < 0.0001), hypertension (PR 1.93, 95% CI 1.68–2.22, *P* < 0.0001) as well as obesity (PR 2.73, 95% CI 2.47–3.02, *P* < 0.0001). Family history of myocardial infarction and/or stroke was similar in both groups (PR_prediabetes_ 1.21, 95% CI 1.09–1.35, *P* = 0.0005; PR_T2DM_ 1.26, 95% CI 1.14–1.40, *P* < 0.0001) (Table 3).

**Table 3:** Interrelation of diabetic phenotypes and traditional cardiovascular risk factors

	Prediabetes (*n* = 13,491)		Diabetes (*n* = 13,382)	
	Prevalence ratio (95% CI)	*P *value	Prevalence ratio (95% CI)	*P *value
Age [10 years]	1.76 (1.67; 1.85)	< 0.0001	1.85 (1.75; 1.96)	< 0.0001
Sex (female)	1.14 (1.04; 1.26)	0.0073	0.76 (0.68; 0.84)	< 0.0001
Dyslipidaemia	1.51 (1.36; 1.66)	< 0.0001	1.88 (1.69; 2.09)	< 0.0001
Family history of MI and/or stroke	1.21 (1.09; 1.35)	0.0005	1.26 (1.14; 1.40)	< 0.0001
Hypertension	1.27 (1.13; 1.42)	< 0.0001	1.93 (1.68; 2.22)	< 0.0001
Obesity	1.78 (1.61; 1.97)	< 0.0001	2.73 (2.47; 3.02)	< 0.0001
Smoking	1.83 (1.63; 2.06)	< 0.0001	1.37 (1.20; 1.58)	< 0.0001

Multiple Poisson regression analysis depicting the prevalence ratio for traditional cardiovascular risk factors as independent variables and diabetic phenotypes as dependent variables (comparator: individuals with euglycaemia)

*CI* confidence interval, *MI* myocardial infarction

Use of the ADA definition of prediabetes revealed comparable results with raising prevalences of cardiovascular risk factors and comorbidities from euglycaemia to prediabetes to T2DM (Supplemental Table S4). Also, the results of Poisson regression analyses were about equal (Supplemental Table S5).

### Diabetic phenotype, subclinical and clinical cardiovascular disease

**Fig. 2:**
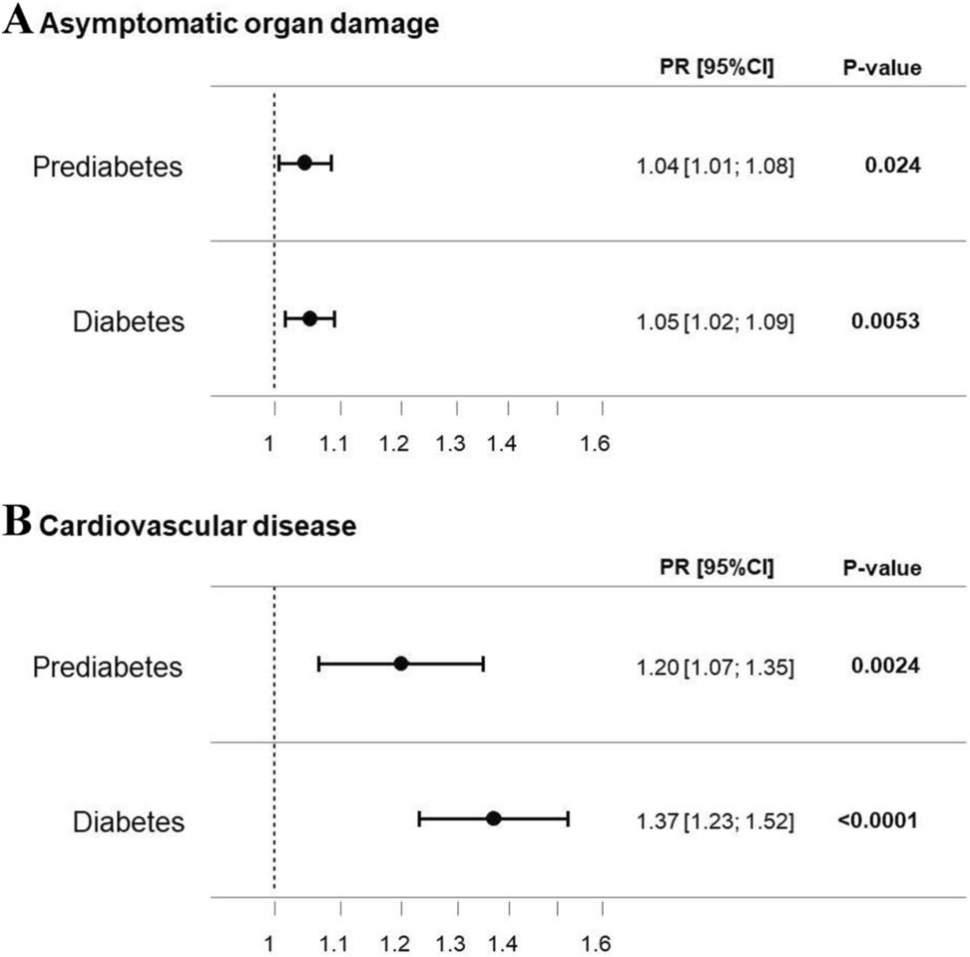
Forest plot illustrating the interrelation of prediabetes and T2DM on asymptomatic organ damage as well as (sub)clinical cardiovascular disease. Graphical illustration of estimates of multiple Poisson regression analysis with asymptomatic cardiovascular organ damage as well as cardiovascular disease as dependent variables and the independent variables prediabetes and T2DM (vs. euglycaemia) adjusted for age, sex, and cardiovascular risk factors

Given the high co-prevalence of both, prediabetes and T2DM, respectively, with traditional CVRF, the relationship between glucose status and CVD was analysed. In multiple Poisson regression analyses with adjustment for age, sex, and traditional CVRF (except T2DM), prediabetes was related to 4% and T2DM with 5% elevated prevalence of asymptomatic cardiovascular organ damage (PR_prediabetes_ 1.04, 95% CI 1.01–1.08, *P* = 0.025; PR_T2DM_ 1.05, 95% CI 1.02–1.09, *P* = 0.005). The prevalence for clinically established CVD was increased by 20% in people with prediabetes and by 37% in individuals with T2DM (PR_prediabetes_ 1.20, 95% CI 1.07–1.35, *P* = 0.002; PR_T2DM_ 1.37, 95% CI 1.23–1.52, *P* < 0.0001) (Fig. 2).

No considerable difference to these results was found after calculation with the ADA definition of prediabetes (AOD: PR_prediabetes_ 1.05, 95% CI 1.02–1.08, *P* = 0.0014; PR_T2DM_ 1.07, 95% CI 1.03–1.11, *P* = 0.0007 and CVD: PR_prediabetes_ 1.22, 95% CI 1.11–1.35, *P* < 0.0001; PR_T2DM_ 1.46, 95% CI 1.30–1.64, *P* < 0.0001).

### Cardiovascular risk and mortality

The 10-year risk for incident coronary heart disease calculated with the Framingham risk score was 11.7 ± 10.3% for euglycaemic individuals. Prediabetes was associated with a 56% elevated risk (18.3 ± 11.9%) and the risk of subjects with T2DM was more than tripled (35.3 ± 18.3) compared to euglycaemic people (Supplemental Figure S1). In the present risk score calculation only individuals without manifest CVD were included.

**Fig. 3:**
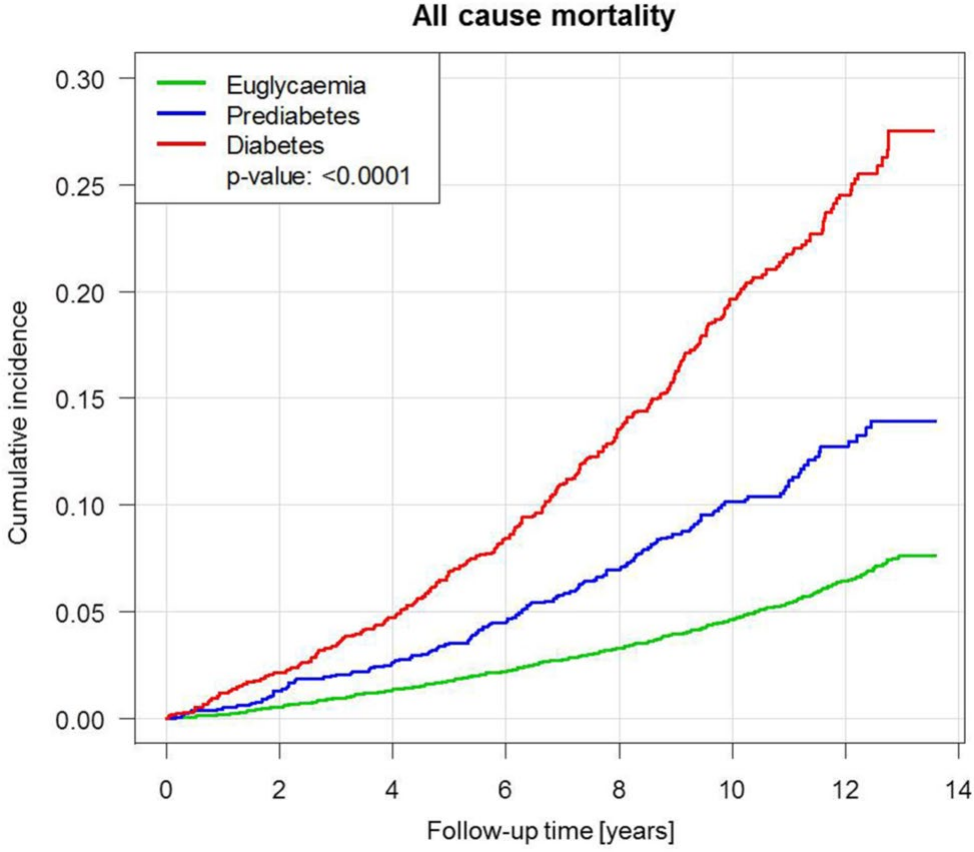
Cumulative incidence of all-cause mortality by diabetic phenotypes in Cox regression analysis. Compared to euglycaemic state, individuals with prediabetes and type 2 diabetes mellitus experienced an elevated risk for death. In the figure, the *P*-value of the log-rank test is provided

**Fig. 4:**
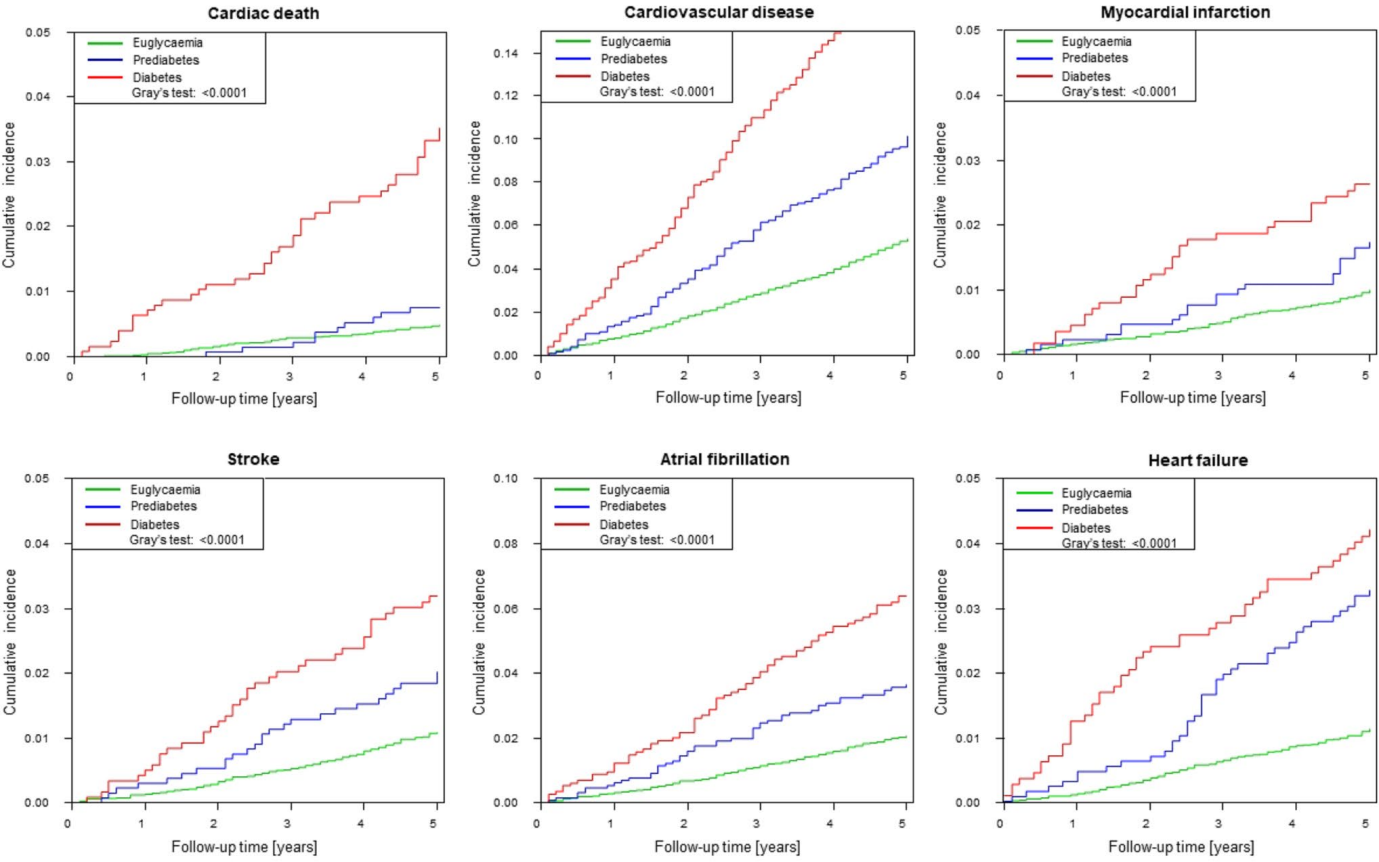
Competing risk analyses for euglycaemia, prediabetes and T2DM with all-cause death as competing risk investigating cardiac death, cardiovascular disease, myocardial infarction, stroke, atrial fibrillation and heart failure. *P *value of Gray’s test is provided

**Fig. 5:**
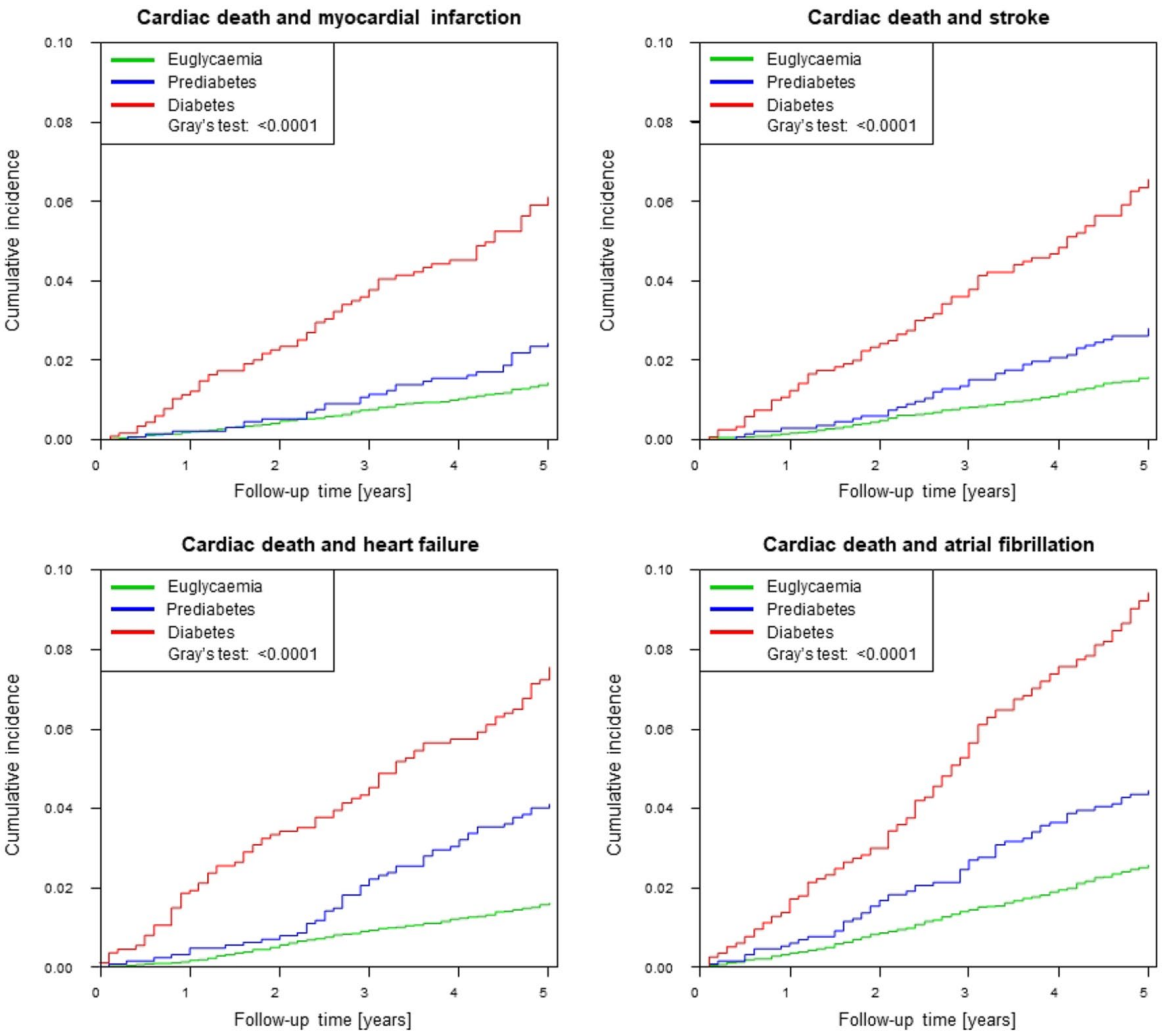
Competing risk analyses for euglycaemia, prediabetes and T2DM with all-cause death as competing risk investigating the combinations of cardiac death and myocardial infarction, cardiac death and stroke, cardiac death and heart failure as well as cardiac death and atrial fibrillation. *P* value of Gray’s test is provided

With view to the high burden of CVRF and subclinical CVD in precursors of T2DM, mortality of prediabetes and T2DM was investigated with Cox regression models and competing risk analyses. For investigation of all-cause mortality using Cox regression during a maximum follow-up time of 13.6 years (median observation time: 10.7 years, IQR 9.32–12.2 years), a total of 1105 deaths were registered. Risk for death from any cause was more than doubled in individuals with prediabetes (hazard ratio (HR) 2.10, 95% CI 1.76–2.51, *P* < 0.0001) and more than fourfold increased in subjects with T2DM (HR 4.28, 95% CI 3.73–4.92, *P* < 0.0001) in comparison to euglycaemic individuals. After adjustment for age and sex, in Cox regression analysis both entities remained independent predictors of death (HR_prediabetes_ 1.30, 95% CI 1.09–1.55, *P* = 0.0038; HR_T2DM_ 2.16, 95% CI 1.88–2.50, *P* < 0.0001), whereas, after additional adjustment for CVRF solely T2DM remained an independent predictor for all-cause mortality (HR 1.89. 95% CI 1.63–2.20, *P* < 0.0001) (Table 4A and Fig. 3). In competing risk analyses T2DM presented after adjustment for sex, age and CVRF (except diabetes) as relevant risk factor for cardiac death (HR 2.60, 95% CI 1.70–3.98, *P* < 0.0001), cardiovascular disease (HR 1.46, 95% CI 1.22–1.75, *P* < 0.0001), heart failure (HR 1.47, 95% CI 1.02–2.12, *P* = 0.042) and atrial fibrillation (HR 1.43, 95% CI 1.07–1.91, *P* = 0.016) as well as for the combinations cardiac death and myocardial infarction (HR 1.73, 95% CI 1.27–2.37, *P* = 0.0006), cardiac death and heart failure (HR 1.72, 95% CI 1.30–2.28, *P* = 0.0002), cardiac death and stroke (HR 1.71, 95% CI 1.28–2.28, *P* = 0.0003), cardiac death and atrial fibrillation (HR 1.58, 95% CI 1.24–2.00, *P* = 0.0006) as well as cardiac death and venous thromboembolism (HR 1.45, 95% CI 1.04–2.04, *P* = 0.03). Prediabetes was found to be a relevant risk factor for heart failure (HR 1.53, 95% CI 1.06–2.20, *P* = 0.023) after adjustment for sex, age and CVRF (except diabetes) (Table 4b, Figs. 4 and 5).

**Table 4:** Multivariable Cox-regression and competing risk analyses of prediabetes and type 2 diabetes mellitus

	Model 1: crude analysis		Model 2: age, sex		Model 3: add. traditional CVRF	
	Hazard ratio (95% CI)	*P* value	Hazard ratio (95% CI)	*P *value	Hazard ratio (95% CI)	*P *value
A. Multivariable Cox regression analysis for all-cause mortality						
Prediabetes	2.10 (1.76; 2.51)	< 0.0001	1.30 (1.09; 1.55)	0.0038	1.17 (0.98; 1.40)	0.09
Diabetes	4.28 (3.73; 4.92)	< 0.0001	2.16 (1.88; 2.50)	< 0.0001	1.89 (1.63; 2.20)	< 0.0001

	Hazard ratio (95% CI)	*P* value	Hazard ratio (95% CI)	*P* value	Hazard ratio (95% CI)	*P* value
B. Competing risk analyses of prediabetes and type 2 diabetes mellitus with all-cause death as competing risk						
Cardiac death						
Prediabetes	1.529 (0.781; 2.995)	0.02	0.907 (0.455; 1.810)	0.78	0.752 (0.379; 1.492)	0.41
Diabetes	7.275 (4.875; 10.858)	< 0.0001	3.434 (2.263; 5.212)	< 0.0001	2.599 (1.697; 3.979)	< 0.0001
Cardiovascular disease						
Prediabetes	1.919 (1.574; 2.339)	< 0.0001	1.189 (0.971; 1.455)	0.09	1.021 (0.833; 1.252)	0.84
Diabetes	3.835 (3.250; 4.525)	< 0.0001	1.878 (1.579; 2.234)	< 0.0001	1.458 (1.217; 1.746)	< 0.0001
Myocardial infarction						
Prediabetes	1.744 (1.105; 2.754)	0.02	1.296 (0.808; 2.079)	0.28	1.047 (0.647; 1.693)	0.85
Diabetes	2.711 (1.802; 4.079)	< 0.0001	1.602 (1.030; 2.490)	0.04	1.233 (0.780; 1.949)	0.37
Heart failure						
Prediabetes	2.937 (2.064; 4.178)	< 0.0001	1.875 (1.299; 2.707)	0.0008	1.528 (1.061; 2.201)	0.023
Diabetes	3.857 (2.748; 5.412)	< 0.0001	2.142 (1.505; 3.050)	< 0.0001	1.465 (1.015; 2.116)	0.042
Stroke						
Prediabetes	1.850 (1.212; 2.825)	0.0044	1.162 (0.757; 1.783)	0.49	1.069 (0.700; 1.631)	0.76
Diabetes	3.005 (2.081; 4.340)	< 0.0001	1.526 (1.042; 2.236)	0.03	1.256 (0.841; 1.877)	0.27
Atrial fibrillation						
Prediabetes	1.785 (1.305; 2.443)	0.0003	1.123 (0.818; 1.542)	0.47	1.050 (0.761; 1.448)	0.77
Diabetes	3.158 (2.420; 4.119)	< 0.0001	1.644 (1.247; 2.166)	0.0004	1.427 (1.068; 1.905)	0.016
Cardiac death and myocardial infarction						
Prediabetes	1.694 (1.154; 2.486)	0.0071	1.149 (0.772; 1.708)	0.49	0.923 (0.619; 1.377)	0.70
Diabetes	4.377 (3.297; 5.812)	< 0.0001	2.342 (1.734; 3.164)	< 0.0001	1.731 (1.265; 2.369)	0.0006
Cardiac death and heart failure						
Prediabetes	2.560 (1.876; 3.493)	< 0.0001	1.579 (1.143; 2.181)	0.0056	1.278 (0.927; 1.762)	0.13
Diabetes	4.849 (3.735; 6.296)	< 0.0001	2.501 (1.905; 3.284)	< 0.0001	1.723 (1.301; 2.282)	0.0002
Cardiac death and stroke						
Prediabetes	1.765 (1.234; 2.525)	0.0019	1.083 (0.752; 1.560)	0.67	0.954 (0.665; 1.368)	0.80
Diabetes	4.297 (3.285; 5.622)	< 0.0001	2.116 (1.601; 2.796)	< 0.0001	1.705 (1.278; 2.275)	0.0003
Cardiac death and atrial fibrillation						
Prediabetes	1.749 (1.316; 2.324)	0.0001	1.079 (0.808; 1.440)	0.61	0.972 (0.726; 1.301)	0.85
Diabetes	3.797 (3.039; 4.745)	< 0.0001	1.903 (1.510; 2.398)	< 0.0001	1.577 (1.241; 2.003)	0.0002
Cardiac death and venous thromboembolism						
Prediabetes	1.916 (1.300; 2.823)	0.001	1.242 (0.833; 1.852)	0.29	1.023 (0.683; 1.531)	0.91
Diabetes	3.516 (2.548; 4.850)	< 0.0001	1.948 (1.402; 2.705)	< 0.0001	1.452 (1.036; 2.035)	0.03

A. Cox regression models to investigate the impact of prediabetes and type 2 diabetes mellitus on all-cause mortality. B. Competing risk analyses of prediabetes and type 2 diabetes mellitus with all-cause death as competing risk. Both analyses were performed by the following models: Model 1 crude analysis; Model 2 adjusted for sex and age; Model 3 adjusted for sex, age, hypertension, dyslipidaemia, obesity, smoking, family history for myocardial infarction or stroke

*CI* confidence interval

Calculation using the ADA definition of prediabetes revealed only few marginal differences in concern of mortality. Framingham risk score assessment was about even (euglycaemia 10.7 ± 9.91%; prediabetes 16.6 ± 11.5%; T2DM 35.3 ± 18.3) and congruent to before mentioned results also in Cox regression analyses solely T2DM remained an independent predictor for all-cause mortality after adjustment for age, sex and CVRF with an almost identical hazard ratio (HR 1.93 95% CI 1.64–2.26, *P* < 0.0001). Further, results of competing risk analyses revealed no decisive differences except for one distinction: with the ADA definition, prediabetes was not identified as relevant risk factor for heart failure after adjustment for sex, age and CVRF (Supplemental Table S6).

## Discussion

The present study provides estimates for the prevalence of prediabetes and T2DM in the general population accompanied by a comprehensive analysis of the associated cardiovascular burden in prediabetic phenotypes. In a large European cohort this highly standardised population-based study demonstrates the early developed cardiovascular burden observed already in individuals with prediabetes compared to euglycaemic individuals. Prediabetes was associated with an increased risk for AOD and incident CVD. In contrast to T2DM, prediabetes was no independent predictor of all-cause mortality in the general population.

It is well-known that regional differences in the prevalence of CVRF and especially in T2DM exist. For example, in Germany estimates reported prevalences of T2DM ranging between 5.8% in Southern Germany and 12% in North-Eastern Germany [14]. Against this background, the prevalence for T2DM of 7.8% in the current study is in line with findings from other population-based studies [15]. Of interest, this prevalence of T2DM weighted for the population of Germany represents the first reported results for Mid-Western Germany. For prediabetes, a lower prevalence was detected in the present study in comparison to other studies. In North-Eastern Germany, the prevalence of prediabetes was estimated to be up to 43.1%, while for Southern Germany a prevalence of prediabetes of 30.1% has been reported [16]. In this context of regional differences in the prevalence of T2DM, it has to be suggested that regional variations may also be present in the setting of prediabetes. Another potential explanation for differences between prevalence estimates is that prediabetes is not uniformly defined across studies and scientific societies. It is known that differing definitions of institutions like ADA and WHO are currently available and used for diabetes mellitus research, which led to the claim by experts for a uniform definition of diabetic phenotypes [3].

Pathophysiologically, the development of T2DM is currently understood as a continuous process with multiple steps ranging from elevated insulin secretion under euglycaemic conditions, followed by increased insulin resistance, decreasing β-cell mass, and breakdown of the gluco-homeostasis [17]. Complementary, the inflammatory and immune response is gradually altered between euglycaemia, prediabetes, and T2DM [18]. However, it is still not fully understood how stages of glycaemic metabolism derailment are taken in each individual, since certain individuals may remain for an indefinite time in a preliminary stage without developing T2DM, while others immediately experience fulminant T2DM. Also prediabetes constitutes an entity with high risk for T2DM, but can persist life-long without the development of diabetes. When considering the development of T2DM as a multi-step pathophysiological model, it seems especially important from a cardiovascular perspective to decipher the premature cardiovascular burden which is present already in prediabetes. Congruent to the results of the present study, clustering of traditional CVRF in prediabetes has shown interrelations with arterial hypertension, dyslipidaemia, and obesity [4, 19–21]. The underlying mechanisms of prediabetes include insulin resistance and dysfunction of beta-cells which induce a pro-inflammatory state, lipolysis, endothelial dysfunction and arterial stiffness [7, 18, 22]. These alterations lead to cardiovascular disease including coronary artery disease with its acute manifestation of myocardial infarction, congestive heart failure, and peripheral vascular disease [22]. In the present study, prediabetes was an independent risk factor for clinically-prevalent cardiovascular disease and comparable asymptomatic cardiovascular organ damage was observed in individuals with prediabetes and individuals with T2DM. This supports the concept that already prediabetes has a considerable clinical impact, at least from a cardiovascular perspective. The vast cardiovascular burden of prediabetes was recently underlined by a meta-analysis including 129 studies [23]. From a pathophysiological point of view, not only glucolipotoxicty and its consequences may be responsible for the early cardiovascular organ damage observed [24]. Data indicate that hyperinsulinaemia and prediabetes are associated with oxidative stress by dysregulation of NO synthesis and eNOS uncoupling leading to endothelial dysfunction, reduced hepatic glucose homeostasis due to reduction of insulin sensitivity and tyrosine nitration of the insulin receptor [24–26]. In parallel, autonomic nerve dysfunction and alterations in the coagulation system were found to be associated with prediabetes [27, 28]. However, prediabetes and T2DM do not portray two distinct ailments but rather have to be understood as conglomerations of various subphenotypes with widely differing clinical risk patterns [29, 30]. In the present study, HbA1c values of two recent definitions for prediabetes revealed comparable results regarding risk for morbidity and mortality. Yet, prediction of metabolic progression is not reflected by current definitions of prediabetes [29] and also T2DM is highly heterogeneous [30]. Cluster analyses revealed significantly differing patient subgroups within the hypernyms of prediabetes and T2DM regarding clinical phenotype, progress of the metabolic derailment and risk for diabetes-associated complications. Subphenotyping of patients with prediabetes and T2DM may improve risk estimation and optimise early target treatment, probably inducing precision medicine to people with glucometabolic disorder. Further focus on cluster analyses are crucial to further decipher the recent heterogeneity of prediabetes and T2DM [29, 30].

For public health management, it is of particular concern that already prediabetes indicates for a deterioration of clinical outcome as reflected by the increased prevalence of asymptomatic organ damage and cardiovascular disease as well as the elevated calculated 10-year risk for incident coronary artery disease. In the setting of secondary prevention following acute myocardial infarction, prediabetes was associated with increased mortality, recurrent myocardial infarction and repeat revascularisation [31]. Therefore, the findings of the present study from the general population stress the need for early prevention efforts in the management of T2DM. Since prediabetes was independently associated with subclinical and clinically-prevalent CVD and subsequent clinical outcome, preventive efforts in the setting of T2DM may need to start far earlier than in the setting of T2DM. Whether this has also implications for the onset of other non-CVD or early complications of T2DM needs to be further investigated.

## Strengths and limitations

The strength of the present study is the highly-standardised clinical and metabolic phenotyping of a large population (> 15,000 individuals). In this setting, the clinical impact of prediabetes was accurately investigated and comparatively confronted with euglycaemia and T2DM in a large population-based study. Due to the recent existence of two HbA1c based definitions of prediabetes (HbA1c window between 6.0 and 6.4% vs. the range between 5.7% and 6.4%) it can be discussed whether the used range in the present study represents euglycaemia with HbA1c levels < 6.0%. However, the results were verified by subsequent analyses using the HbA1c window between 5.7 and 6.4%, which revealed comparable results, as can be seen in the data provided in the Supplemental material. Due to the nature of the study setting, extrapolation of the findings to other ethnicities has to be done with caution, as well as to cohorts with varying age ranges. Future studies will be necessary to investigate the long-term outcome of prediabetes compared to euglycaemia und T2DM with regard to specific causes of death and prospective investigation of incident cardiovascular events. However, it is striking that prediabetes was already a strong predictor of subclinical and clinically-prevalent CVD in this study independent of established risk factors indicating a high clinical relevance in the general population.

## Conclusions

Besides T2DM, also prediabetes inherits a significant cardiovascular burden, which translates into poor clinical outcome. The present study elucidates the complex interplay between different diabetic phenotypes and their associated clinical and subclinical cardiovascular organ damage and clinical outcome. The study reiterates the need for harmonisation of the definition of prediabetes, which will facilitate future research efforts. The high cardiovascular burden of individuals with prediabetes calls for novel strategies in the primary prevention of T2DM. Early preventive efforts may represent a strong tool to curb the epidemic of T2DM and its associated cardiovascular morbidity.

## Supplementary Information

Below is the link to the electronic supplementary material.

Supplementary file1 (DOCX 111 kb)

## Abbreviations

ADA: American Diabetes Association
AOD: Asymptomatic organ damage
CI: Confidence interval
CVD: Cardiovascular disease
CVRF: Cardiovascular risk factor(s)
FH of MI/stroke: Family history of myocardial infarction or stroke
GHS: Gutenberg Health Study
HR: Hazard ratio
IQR: Interquartile range
PR: Prevalence ratio
SD: Standard deviation
T2DM: Type 2 diabetes mellitus
vs.: Versus
