# Supplementary material for: Cardiovascular profiling in the diabetic continuum: results from the population-based Gutenberg Health Study
Source: Clin Res Cardiol. 2021 Jun 24;111(3):272–83. doi: 10.1007/s00392-021-01879-y (PMC8873120; doi:10.1007/s00392-021-01879-y)
Supplement: Supplementary file 1 — Supplementary file1 (DOCX 111 kb) [file 392_2021_1879_MOESM1_ESM.docx]

**SUPPLEMENTAL APPENDIX**

**Cardiovascular profiling in the diabetic continuum –**

**Results from the population-based Gutenberg Health Study**

Volker H. Schmitt^1,2^, Anja Leuschner^1^, Claus Jünger^7^, Antonio Pinto^3^, Omar Hahad^1,2^, Andreas Schulz^3,4^, Natalie Arnold^3,5,^, Sven-Oliver Tröbs^1,2,3^, Marina Panova-Noeva^4^, Karsten Keller^1,4,11^, Tanja Zeller^5,6^, Manfred Beutel^7^, Norbert Pfeiffer^8^, Konstantin Strauch^9^, Stefan Blankenberg^5,6^, Karl J. Lackner^10,2^, Jürgen H. Prochaska^1,2,3,4*^, Philipp S. Wild ^1,2,3,4*^, Thomas Münzel^1,2,4*^

^1^Department of Cardiology – Cardiology I, University Medical Center, Johannes Gutenberg University Mainz, Langenbeckstr. 1, 55131 Mainz, Germany; ^2^German Center for Cardiovascular Research (DZHK), Partner Site Rhine-Main, Mainz, Germany; ^3^Preventive Cardiology and Preventive Medicine – Department of Cardiology, University Medical Center, Johannes Gutenberg University Mainz, Langenbeckstr. 1, 55131 Mainz, Germany; ^4^Center for Thrombosis and Hemostasis (CTH), University Medical Center, Johannes Gutenberg University Mainz, Langenbeckstr. 1, 55131 Mainz, Germany; ^5^Department of General and Interventional Cardiology, University Heart Center Hamburg, Martinistr 52, 20246 Hamburg, Germany; ^6^German Center for Cardiovascular Research (DZHK), Partner Site Hamburg/Kiel/Lübeck, Hamburg, Germany; ^7^Department of Psychosomatic Medicine and Psychotherapy, University Medical Center, Johannes Gutenberg University Mainz, Langenbeckstr. 1, 55131 Mainz, Germany; ^8^Department of Ophthalmology, University Medical Center, Johannes Gutenberg University Mainz, Langenbeckstr. 1, 55131 Mainz, Germany; ^9^Institute for Medical Biometrics, Epidemiology and Informatics (IMBEI), University Medical Center, Johannes Gutenberg University Mainz, Obere Zahlbacher Str. 69, 55131 Mainz, Germany; ^10^Institute of Clinical Chemistry and Laboratory Medicine, University Medical Center, Johannes Gutenberg University Mainz, Langenbeckstr. 1, 55131 Mainz, Germany; ^11^Medical Clinic VII, Department of Sports Medicine, University Hospital Heidelberg, Heidelberg, Germany

*contributed equally.

**Supplemental Table 1.** Definition of cardiovascular risk factors.

| **Risk factor** | **Definition** |
| --- | --- |
| Arterial hypertension | systolic blood pressure ≥ 140 mmHg or  diastolic blood pressure ≥ 90 mmHg or  hypertension diagnosed by a physician or  medication treatment of hypertension (self-reported) |
| Dyslipidaemia | LDL/HDL-ratio >3.5 or  dyslipidaemia diagnosed by a physician or  medication treatment of dyslipidaemia (self-reported) |
| Obesity | BMI ≥ 30 |
| Smoking status | currently active smoking or  no currently active smoking (= no smoker) |
| Positive family history of disease | at least one reported first degree relative with  - myocardial infarction or  - stroke at an age ≤ 60 years (male) or ≤ 65 years (female) |
| BMI, Body mass index; HDL, high density lipoprotein; LDL, low density lipoprotein. | |

**Supplemental Table 2.** Definition of asymptomatic cardiovascular organ damage and cardiovascular disease**.**

| **Cardiovascular phenotype** | **Definition** |
| --- | --- |
| Asymptomatic cardiovascular organ damage | no prevalent cardiovascular disease;  elevated left ventricular mass index (men >115 g/m², women >95 g/m²) or  intima-media-thickness >0.9 mm or  presence of at least one atherosclerotic plaque in the carotid artery or  ankle-brachial-index <0.9 or  renal insufficiency (glomerular filtration rate 30-60 ml/min/1.73m²) or  microalbuminuria (albumin-creatinine-ratio 30-300 mg/g) |
| Cardiovascular disease | coronary artery disease or  myocardial infarction or  stroke or transient ischemic attack or  heart failure or  atrial fibrillation or  peripheral artery disease |
| Table showing the definitions of cardiovascular disease and asymptomatic organ damage according to the ESC guidelines on cardiovascular disease prevention in clinical practice [[1](#_ENREF_1)]. | |

**Supplemental Table 3. Prevalence of euglycaemia, prediabetes (defined as HbA1c 5.7-6.4%) and T2DM according to age within the GHS and region of residence.**

| **A. Prevalence of prediabetes and T2DM according to age decades in the GHS study cohort** | | | | |
| --- | --- | --- | --- | --- |
|  | **35-44 years** | **45-54 years** | **55-64 years** | **65-74 years** |
| Euglycaemia | 87.8% (*n* = 2,863) | 71.6% (*n* = 2,825) | 54.4% (*n* = 2,145) | 42.8% (*n* = 1,593) |
| Prediabetes | 10.9% (*n* = 354) | 24.1% (*n* = 951) | 34.3% (*n* = 1,351) | 39.6% (*n* = 1,472) |
| Diabetes | 1.3% (*n* = 44) | 4.3% (*n* = 171) | 11.3% (*n* = 447) | 17.7% (*n* = 654) |
|  |  | | | |
| **B. Prevalence of prediabetes and T2DM in the GHS study sample and weighted for the population of Mainz/Mainz-Bingen, the population of Germany and the European standard population of 1976** | | | | |
|  | **GHS Study sample** | **Population of Mainz and**  **Mainz-Bingen, Germany** | **Population of Germany** | **European standard**  **population** |
| Euglycaemia | 63.4% (*n* = 9,426) | 67.2% (*n* = 9,994) | 66.2% (*n* = 9,843) | 68.3% (*n* = 10,156) |
| Prediabetes | 27.8% (*n* = 4,128) | 25.4% (*n* = 3,776) | 26.1% (*n* = 3,875) | 24.7% (*n* = 3,679) |
| Diabetes | 8.9% (*n* = 1,316) | 7.4% (*n* = 1,102) | 7.8% (*n* = 1,154) | 7.0% (*n* = 1,037) |
| A. Presentation of the prevalence estimates of euglycaemia, prediabetes and T2DM according to age decades in the GHS study sample. B. Table showing prevalences of euglycaemia, prediabetes and T2DM as unweighted data for the GHS study sample and weighted data (for age- and sex-distribution) for the population of Mainz/Mainz-Bingen, Germany and the European standard population of 1976. Prevalence estimates are provided as relative (%) and absolute (*n*) frequency. GHS: Gutenberg Health Study. T2DM: type 2 diabetes mellitus. | | | | |

**Supplemental Table 4. Characteristics of study participants according to diabetic status (prediabetes defined as HbA1c 5.7-6.4%).**

|  | **Euglycaemia**  **(*n* = 9,429)** | **Prediabetes**  **(*n* = 4,128)** | **Diabetes**  **(*n* = 1,316)** | **P-value** |
| --- | --- | --- | --- | --- |
| Age, mean (SD), years | 52.0±10.8 | 59.3±9.7 | 63.0±8.3 | <0.0001 |
| Female sex | 50.8% (4,784) | 50.0% (2,065) | 38.2% (503) | <0.0001 |
| BMI, median (IQR), kg/m² | 25.9 (23.3/28.9) | 27.5 (24.7/30.9) | 30.7 (27.3/34.6) | <0.0001 |
| *Traditional cardiovascular risk factors* | | | | |
| Current smoking | 19.1% (1,802) | 21.2% (874) | 16.1% (210) | 0.61 |
| Dyslipidaemia | 26.8% (2,526) | 43.6% (1,796) | 61.8% (810) | <0.0001 |
| Family history of MI and/or stroke | 20.7% (1,949) | 23.6% (975) | 27.5% (362) | <0.0001 |
| Hypertension | 41.2% (3,883) | 59.4% (2,451) | 80.2% (1,056) | <0.0001 |
| Obesity | 18.6% (1,755) | 30.6% (1,262) | 55.3% (727) | <0.0001 |
| *Cardiovascular comorbidities* | | | | |
| Atrial fibrillation | 1.9% (180) | 3.6% (148) | 5.8% (76) | <0.0001 |
| Congestive heart failure | 0.8% (77) | 1.8% (75) | 3.4% (44) | <0.0001 |
| Coronary artery disease | 2.2% (210) | 6.0% (247) | 13.5% (177) | <0.0001 |
| Myocardial infarction | 1.5% (140) | 4.2% (173) | 9.5% (125) | <0.0001 |
| Peripheral artery disease | 2.1% (202) | 4.5% (184) | 8.1% (107) | <0.0001 |
| Stroke | 1.2% (113) | 2.3% (96) | 4.9% (64) | <0.0001 |
| *Laboratory parameters of glucose metabolism* | | | | |
| Glucose, median (IQR), mg/dl | 89.0 (84.0/95.0) | 94.2 (89.0/101.0) | 112.2 (99.0/131.2) | <0.0001 |
| HbA1c, median (IQR), % | 5.30 (5.10/5.50) | 5.90 (5.70/6.00) | 6.70 (6.30/7.20) | <0.0001 |
| Discrete variables are expressed as relative and absolute frequencies; continuous variables are provided according to distribution as mean with standard deviation or median with interquartile range. BMI, body mass index; HbA1c, glycated haemoglobin; IQR, interquartile range; MI, myocardial infarction; SD, standard deviation. | | | | |

**Supplemental Table 5. Interrelation of diabetic phenotypes and traditional cardiovascular risk factors (prediabetes defined as HbA1c 5.7-6.4%).**

|  | **Prediabetes**  **(*n* = 13,508)** | | **Diabetes**  **(*n* = 10,697)** | |
| --- | --- | --- | --- | --- |
|  | Prevalence ratio  (95% CI) | P-value | Prevalence ratio  (95% CI) | P*-*value |
| Age [10 years] | 1.50 (1.46; 1.54) | <0.0001 | 1.92 (1.82; 2.03) | <0.0001 |
| Sex (female) | 1.04 (0.99; 1.09) | 0.1 | 0.81 (0.73; 0.87) | <0.0001 |
| Dyslipidaemia | 1.31 (1.24; 1.38) | <0.0001 | 1.92 (1.73; 2.13) | <0.0001 |
| Family history of MI and/or stroke | 1.07 (1.01; 1.14) | 0.014 | 1.23 (1.11; 1.36) | <0.0001 |
| Hypertension | 1.12 (1.06; 1.19) | <0.0001 | 1.92 (1.67; 2.20) | <0.0001 |
| Obesity | 1.37 (1.30; 1.44) | <0.0001 | 2.61 (2.36; 2.87) | <0.0001 |
| Smoking | 1.38 (1.30; 1.47) | <0.0001 | 1.43 (1.25; 1.63) | <0.0001 |
| Multiple Poisson regression analysis depicting the prevalence ratio for traditional cardiovascular risk factors as independent variables and diabetic phenotypes as dependent variables (comparator: individuals with euglycaemia). CI, confidence interval; MI, myocardial infarction. | | | | |

**Supplemental Table 6. Multivariable Cox-regression and competing risk analyses of prediabetes (defined as HbA1c 5.7-6.4%) and type 2 diabetes mellitus.**

|  | **Model 1: Crude analysis** | | **Model 2: age, sex** | | **Model 3: add. traditional CVRF** | |
| --- | --- | --- | --- | --- | --- | --- |
|  | **Hazard ratio (95%CI)** | **P-value** | **Hazard ratio (95%CI)** | **P-value** | **Hazard ratio (95%CI)** | **P-value** |
| **A. Multivariable Cox-regression analysis for all-cause mortality** | | | |  |  |  |
| Prediabetes | 1.99 (1.73; 2.28) | <0.0001 | 1.20 (1.04; 1.38) | 0.01 | 1.11 (0.97; 1.28) | 0.13 |
| Diabetes | 4.95 (4.27; 5.75) | <0.0001 | 2.24 (1.92; 2.61) | <0.0001 | 1.93 (1.64; 2.26) | <0.0001 |
|  |  |  |  |  |  |  |
| **B. Competing risk analyses of prediabetes and type 2 diabetes mellitus with all-cause death as competing risk** | | | | | | |
| **Cardiac death** |  |  |  |  |  |  |
| Prediabetes | 1.92 (1.19; 3.12) | 0.0082 | 1.13 (0.70; 1.85) | 0.63 | 0.99 (0.61; 1.61) | 0.97 |
| Diabetes | 8.83 (5.66; 13.78) | <0.0001 | 3.71 (2.33; 5.90) | <0.0001 | 2.75 (1.72; 4.40) | <0.0001 |
| **Cardiovascular disease** |  |  |  |  |  |  |
| Prediabetes | 1.80 (1.55; 2.01) | <0.0001 | 1.12 (0.96; 1.31) | 0.14 | 1.00 (0.86; 1.17) | 0.99 |
| Diabetes | 4.33 (3.64; 5.15) | <0.0001 | 1.92 (1.60; 2.31) | <0.0001 | 1.46 (1.21; 1.76) | 0.0001 |
| **Myocardial infarction** |  |  |  |  |  |  |
| Prediabetes | 1.87 (1.33; 2.63) | 0.0003 | 1.39 (0.98; 1.99) | 0.067 | 1.18 (0.82; 1.69) | 0.37 |
| Diabetes | 3.18 (2.07; 4.87) | <0.0001 | 1.79 (1.12; 2.85) | 0.015 | 1.34 (0.82; 2.17) | 0.24 |
| **Heart failure** |  |  |  |  |  |  |
| Prediabetes | 2.34 (1.73; 3.18) | <0.0001 | 1.50 (1.09; 2.06) | 0.012 | 1.28 (0.93; 1.77) | 0.12 |
| Diabetes | 4.52 (3.15; 6.48) | <0.0001 | 2.28 (1.56; 3.33) | <0.0001 | 1.51 (1.02; 2.24) | 0.042 |
| **Stroke** |  |  |  |  |  |  |
| Prediabetes | 1.51 (1.09; 2.10) | 0.013 | 0.94 (0.67; 1.31) | 0.70 | 0.87 (0.62; 1.21) | 0.40 |
| Diabetes | 3.17 (2.17; 4.65) | <0.0001 | 1.45 (0.97; 2.16) | 0.071 | 1.15 (0.76; 1.75) | 0.51 |
| **Atrial fibrillation** |  |  |  |  |  |  |
| Prediabetes | 1.63 (1.28; 2.01) | <0.0001 | 1.02 (0.80; 1.30) | 0.88 | 0.96 (0.75; 1.23) | 0.73 |
| Diabetes | 3.47 (2.63; 4.59) | <0.0001 | 1.63 (1.22; 2.18) | 0.001 | 1.39 (1.03; 1.88) | 0.033 |
| **Cardiac death and myocardial infarction** | |  |  |  |  |  |
| Prediabetes | 1.87 (1.41; 2.49) | <0.0001 | 1.27 (0.95; 1.70) | 0.11 | 1.07 (0.80; 1.44) | 0.63 |
| Diabetes | 5.15 (3.80; 6.98) | <0.0001 | 2.56 (1.85; 3.54) | <0.0001 | 1.84 (1.31; 2.57) | 0.0004 |
| **Cardiac death and heart failure** | |  |  |  |  |  |
| Prediabetes | 2.30 (1.77; 2.97) | <0.0001 | 1.42 (1.09; 1.86) | 0.0098 | 1.22 (0.94; 1.60) | 0.14 |
| Diabetes | 5.80 (4.38; 7.69) | <0.0001 | 2.71 (2.02; 3.63) | <0.0001 | 1.81 (1.33; 2.46) | 0.0001 |
| **Cardiac death and stroke** | |  |  |  |  |  |
| Prediabetes | 1.62 (1.24; 2.13) | 0.0005 | 0.99 (0.75; 1.30) | 0.92 | 0.89 (0.68; 1.18) | 0.42 |
| Diabetes | 4.71 (3.54; 6.26) | <0.0001 | 2.08 (1.54; 2.79) | <0.0001 | 1.63 (1.20; 2.21) | 0.0018 |
| **Cardiac death and atrial fibrillation** | |  |  |  |  |  |
| Prediabetes | 1.70 (1.37; 2.11) | <0.0001 | 1.05 (0.84; 1.30) | 0.67 | 0.96 (0.77; 1.20) | 0.74 |
| Diabetes | 4.27 (3.37; 5.40) | <0.0001 | 1.93 (1.51; 2.46) | <0.0001 | 1.57 (1.22; 2.02) | 0.0005 |
| **Cardiac death and venous thromboembolism** | |  |  |  |  |  |
| Prediabetes | 2.06 (1.53; 2.77) | <0.0001 | 1.35 (0.99; 1.83) | 0.058 | 1.20 (0.88; 1.63) | 0.24 |
| Diabetes | 4.23 (3.00; 5.96) | <0.0001 | 2.16 (1.52; 3.07) | <0.0001 | 1.60 (1.12; 2.28) | 0.011 |
| A. Cox regression models to investigate the impact of prediabetes and type 2 diabetes mellitus on all-cause mortality. B. Competing risk analyses of prediabetes and type 2 diabetes mellitus with all-cause death as competing risk. Both analyses were performed by the following models: Model 1 crude analysis; Model 2 adjusted for sex and age; Model 3 adjusted for sex, age, hypertension, dyslipidemia, obesity, smoking, family history for myocardial infarction or stroke. CI: confidence interval. | | | | | | |

**Supplemental Figure 1**

Title: Predicted 10-year risk for incident coronary heart disease

Caption: Individuals with prediabetes revealed a more than 1.5-fold risk for coronary artery disease within 10-years compared to euglycaemic subjects. People with T2DM were associated with a more than threefold 10-year risk for incident coronary heart disease according to Framingham risk score [2] in comparison to euglycaemic individuals.

**
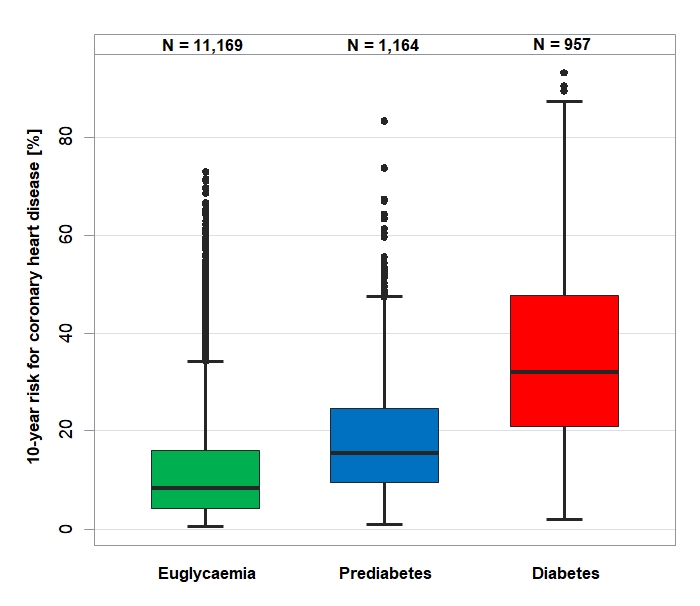
**

**References**

1 Piepoli MF, Hoes AW, Agewall S, et al. (2016) 2016 European Guidelines on cardiovascular disease prevention in clinical practice: The Sixth Joint Task Force of the European Society of Cardiology and Other Societies on Cardiovascular Disease Prevention in Clinical Practice (constituted by representatives of 10 societies and by invited experts)Developed with the special contribution of the European Association for Cardiovascular Prevention & Rehabilitation (EACPR). Eur Heart J 37: 2315-2381. 10.1093/eurheartj/ehw106.

2. D'Agostino RB, Sr., Vasan RS, Pencina MJ, et al. General cardiovascular risk profile for use in primary care: the Framingham Heart Study. Circulation 2008;117(6):743-53
